# Interpreting p‐tau217 on the ward: frailty and plasma biomarkers in acutely admitted older adults

**DOI:** 10.1002/alz.71768

**Published:** 2026-08-21

**Authors:** Jonas Goll, Ieva Martinaityte, Bjørn‐Eivind Kirsebom, Dag Seeger Halvorsen, Fernando Gonzalez‐Ortiz, Henrik Zetterberg, Tormod Fladby, Mona Dixon Gundersen

**Affiliations:** ^1^ Department of Geriatrics University Hospital of North Norway Tromsø Norway; ^2^ Department of Clinical Medicine Faculty of Health Sciences The Arctic University of Norway Tromsø Norway; ^3^ Department of Neurology University Hospital of North Norway Tromsø Norway; ^4^ Department of Psychology Faculty of Health Sciences The Arctic University of Norway Tromsø Norway; ^5^ Department of Neurology Akershus University Hospital Lørenskog Norway; ^6^ Department of Psychiatry and Neurochemistry Institute of Neuroscience and Physiology the Sahlgrenska Academy at the University of Gothenburg Mölndal Sweden; ^7^ Clinical Neurochemistry Laboratory Sahlgrenska University Hospital Mölndal Sweden; ^8^ Department of Pathology and Laboratory Medicine University of Wisconsin School of Medicine and Public Health Madison Wisconsin USA; ^9^ Wisconsin Alzheimer's Disease Research Centre University of Wisconsin School of Medicine and Public Health University of Wisconsin‐Madison Madison Wisconsin USA; ^10^ Department of Neurodegenerative Disease UCL Institute of Neurology London UK; ^11^ UK Dementia Research Institute at UCL London UK; ^12^ Centre for Brain Research Indian Institute of Science Bangalore India

**Keywords:** aging, Alzheimer's disease, blood biomarkers, clinical heterogeneity, cognitive decline, frailty, kidney function, NfL, pathophysiology, plasma, p‐tau217

## Abstract

**INTRODUCTION:**

Older adults living with frailty are prone to adverse events upon acute hospitalization. Frailty biomarkers aiding early identification are lacking. This study explores frailty and Alzheimer's disease (AD)‐related biomarkers.

**METHODS:**

Participants were recruited from the Norfrail study (*n* = 178) and from the Norwegian Dementia Disease Initiative (*n* = 105). Linear regression was used to analyze associations between frailty and the plasma biomarkers phosphorylated tau‐217 (p‐tau217), neurofilament light chain (NfL), brain‐derived tau, ubiquitin carboxyl‐terminal hydrolase L1, and glial fibrillary acidic protein in older adults admitted acutely to hospital.

**RESULTS:**

No significant association was found between p‐tau217 and frailty. Estimated glomerular filtration rate (eGFR) explained 27% of the variance of plasma p‐tau217 (*p* < 0.001). NfL was the only biomarker significantly associated with frailty (*p* < 0.01).

**DISCUSSION:**

Except for NfL, plasma biomarkers were independent of frailty. Substantial p‐tau217 variability was explained by eGFR. Clinicians need to consider renal function when interpreting p‐tau217 in unselected populations.

## Highlights

- AD‐specific plasma biomarkers were not associated with frailty.
- Novelly, brain‐derived tau was independent of frailty.
- Twenty‐seven percent of variance in phosphorylated tau‐217 was explained by kidney dysfunction.
- Only plasma NfL was significantly associated with frailty.

## 1 BACKGROUND

Frailty is an age‐related geriatric syndrome characterized by a decline in physiological reserves, rendering the individual vulnerable to internal and external stressors.^1^ The underlying mechanisms of frailty are multifactorial and include immunosenescence, inflammaging, and sarcopenia.^2^ Several studies propose a bidirectional link between frailty and cognitive decline, although it is not clear which mechanisms are involved.^3^ To date, no biomarkers for frailty have been established. Importantly, frailty is considered a dynamic state with potential reversibility.^4^, ^5^ Early identification of frailty is an integral part of clinical assessment of older adults and key to optimizing acute hospital care.^6^ Several screening tools are validated for use in the emergency department (ED), including the widely used Clinical Frailty Scale (CFS).^7^, ^8^ However, these tools are limited by their ability to differentiate between cognitive impairment and physical frailty.^9^ Recent advances in the field of plasma biomarkers for Alzheimer's disease (AD) have raised questions about whether these biomarkers overlap with or are independent of frailty.

Phosphorylated tau at threonine 217 (p‐tau217) is currently the most promising plasma biomarker of AD neuropathologic change (ADNC)^10^ and is considered a core 1 biomarker in the National Institute on Aging and Alzheimer's Association (NIA‐AA) diagnostic criteria.^11^ To date, plasma p‐tau217 has primarily been evaluated in well‐characterized research cohorts and population‐based studies; however, it is largely unexplored in relation to frailty.^10^ It is not known how brain‐derived tau (BD‐tau), a novel blood‐based biomarker for ADNC, is affected by frailty.^12^

Neurofilament light chain (NfL) is a protein supporting axonal integrity in both the peripheral and central nervous system.^13^ Elevated levels of NfL have been reported in individuals living with frailty, independent of cognitive status,^14^ and may contribute to identify frailty in community dwelling older adults.^15^ Glial fibrillary acidic protein (GFAP) has also been linked with physical frailty, but results are mixed.^16^, ^17^ Ubiquitin carboxyl‐terminal hydrolase L1 (UCHL1) is a biomarker for neuronal injury^18^ that is unexplored in relation to frailty.

The aim of our study was to explore whether AD plasma biomarkers were associated with frailty in a heterogeneous cohort of older adults presenting to the ED. A panel of biomarkers reflecting different pathophysiological mechanisms (NfL, GFAP, BD‐tau, and UCHL1) was included. We investigated relations between the biomarkers and other selected covariates including age, sex, body mass index (BMI), kidney function, and C‐reactive protein (CRP). Finally, a comparison of p‐tau217 levels with a cohort including healthy controls and individuals living with mild cognitive impairment (MCI) was included.

**RESEARCH IN CONTEXT**

1. **Systematic review**: We reviewed the existing literature on plasma biomarkers for frailty and AD. The pathophysiology is poorly understood, and a bidirectional link has been proposed. Results in this field are mixed. Plasma phosphorylated tau at threonine 217 (p‐tau217) is largely unexplored in relation to frailty, and few studies have included “real‐world” cohorts. To our knowledge, BD‐tau and UCHL1 have not been examined in relation to frailty.
2. **Interpretation**: P‐tau217 and BD‐tau showed no association with frailty, suggesting that frailty and AD have different pathophysiological pathways. A large proportion of p‐tau217 variability was explained by eGFR. NfL was the only plasma biomarker associated with frailty.
3. **Future directions**: There is a need for biomarkers that aid early identification of frailty. Incorporating neurocognitive biomarkers in frailty assessment may aid clinicians in distinguishing physical frailty from frailty driven by cognitive decline.

## 2 METHODS

### 2.1 Study population

Two independent cohorts were included from the Norfrail study (cohort 1) and the Norwegian Dementia Disease Initiation (DDI) study (cohort 2). Details are given below.

### 2.2 Cohort 1: Norfrail study

The Norfrail study included older adults aged ≥ 65 years admitted acutely to the ED at the University Hospital of North Norway, Tromsø, in the period January 1, 2025 to July 1, 2025.

Exclusion criteria were outpatient treatment in the ED, patients isolated for infection control, and patients deemed not eligible for the study by their treating physician.

All study participants gave written informed consent. In cases of impaired mental capacity, their guardian was consulted. The project was approved by the regional committee for medical and health research ethics (reference number 628856) and conducted in accordance with the Declaration of Helsinki and the Norwegian Health Research Act.

### 2.3 Baseline evaluation

All participants were assessed at baseline within 24 h of admission by either the research doctor or the research nurse, using the CFS, modified Fried frailty phenotype (mFFP), Charlson Comorbidity Index, and the delirium screening test the 4 A's Test (4AT).^19^, ^20^, ^21^, ^22^ In addition, educational level, history of falls, smoking, medication use, height, and weight were collected. Blood samples were collected between 8:00 a.m. and 2:00 p.m. due to laboratory logistics. Length of hospital stay, ED triage results using the Rapid Emergency Triage and Treatment System, intensive care treatment, ED discharge diagnosis, and clinical variables were obtained from the participants’ medical records.^23^ Estimated glomerular filtration rate (eGFR) was calculated using the eGFRcr21 formula.^24^ Three‐month mortality data were collected through the electronic medical records, which are continuously updated by the Norwegian Cause of Death Registry.

### 2.4 Blood samples

Venous blood samples were drawn by the research personnel at baseline evaluation and immediately transported to the laboratory, with a maximum delay of 30 min, well within the manufacturer's recommendation. Plasma samples were centrifuged at 2000 × g for 15 min immediately upon arrival at the laboratory. After centrifugation, 1‐mL samples of the supernatant were aliquoted in cryotubes and frozen to −20°C before transportation to long‐term storage at −80°C.

### 2.5 Frailty assessment

Frailty was assessed with the CFS by the research doctor or research nurse trained in frailty assessment.^7^ In addition, a mFFP was determined.^20^, ^21^ The original criteria were used for exhaustion (identified by two questions from the Centre for Epidemiologic Studies Depression Scale) and self‐reported unintentional weight loss of 4.5 kg or 5% of body weight in the last year. Grip strength was measured three times using a Jamar hand dynamometer (Jamar 081028935) and averaged on both sides, and the best side was then compared to the original cut‐offs given by Fried et al.^2^ Low physical activity was assessed using the four‐item Lipid Research Clinics questionnaire.^25^ Participants were classified at low physical activity level if they reported not engaging in strenuous exercise and being less active than their peers. Instead of measuring walking speed, participants were asked to describe their walking speed and categorized as having a slow walking speed if they walked slower than their peers or worse.^20^

### 2.6 Cohort 2: DDI

The DDI study cohort includes individuals aged 40 to 80 years, recruited from memory clinics and local advertisements across Norway. The cohort comprises cognitively normal (CN) participants – with or without subjective cognitive decline – and individuals with MCI. All participants undergo standardized diagnostic assessments, including medical history, neurological examination, neuropsychological testing, and collection of cerebrospinal fluid and blood samples. Detailed cohort characteristics and diagnostic criteria have been described previously.^26^ In the present study, however, CN versus MCI was defined using an actuarial approach based solely on neuropsychological performance, without requiring the presence of subjective cognitive symptoms, as described in our previous work.^27^ A total of 105 participants from the DDI cohort were included. Details on the selection are given in Appendix A.

### 2.7 Plasma biomarker measurements

Plasma biomarkers were analyzed on the Simoa HD‐X platform at the University of Gothenburg. Plasma p‐tau217 in the Norfrail and DDI cohorts was measured using a previously validated method.^28^ Plasma BD‐tau, GFAP, UCHL1, and NfL in the Norfrail cohort were measured using commercially available kits (Neurology 4‐Plex D, product no.: 104822). Plasma biomarkers were measured with four‐fold dilution factor and intra‐ and interplate variability was controlled using internal quality controls with a coefficient of variance of <15% for all analytes.

In the DDI cohort, cerebrospinal fluid Aβ_1‐42_ and Aβ_1‐40_ concentrations were measured using the QuickPlex SQ 120 system (Meso Scale Discovery, Rockville, MD, USA) in a multiplex format with the V‐PLEX Aβ Peptide Panel 1 (6E10) kit (K15200E‐1). The cut‐off for the cerebrospinal fluid Aβ_42/40_ ratio (≤0.077) was established based on receiver operating characteristic (ROC) analysis, using visual reads of [^1^ ^8^F]‐flutemetamol positron emission tomography scans as the reference standard.^29^

### 2.8 Statistical analysis

All analyses were performed in RStudio (R version 4.3.2). An analysis of variance (ANOVA) was performed to assess between‐group differences in age and plasma p‐tau217 levels between the Norfrail sample and cerebrospinal fluid Aβ_42/40_ ratio defined amyloid positive (A+) or negative (A−) groups from the DDI cohort. Post hoc comparisons between respective groups were performed without adjustment for multiple comparisons. A chi‐squared test was performed to assess differences in sex distribution. Linear regression models were fitted to examine associations between the CFS and mFFP scores (independent variables) and each plasma biomarker (dependent variable). All biomarker measures were log‐transformed, and all continuous variables, including covariates, were standardized prior to analysis. Covariates (age, sex, eGFR, CRP, and BMI) were evaluated using hierarchical regression, with entry order determined by assumed theoretical relevance. Because the eGFR formula incorporates age, covariates were entered sequentially as follows: eGFR, age, BMI, sex, and CRP. Model blocks were compared using ANOVA and entered stepwise; covariates were retained in the final model if they both explained additional variance and improved model fit based on the Bayesian information criterion (BIC). For CFS and mFFP, *p* values were adjusted across biomarker models using the Benjamini–Hochberg (BH) procedure. Partial coefficients of determination (*R* ^2^_p_) were reported for statistically significant predictors, reflecting the proportion of variance uniquely explained by each term. As the number of available biomarker observations varied (*n* = 164 to 169), additional sensitivity analyses were conducted on the subset with complete data for all markers (*n* = 158). Model assumptions – including linearity, homoscedasticity, normality of residuals, independence of errors, multicollinearity, and influential observations – were assessed using diagnostic plots and indices from the performance R package.^30^ This included visual inspection of residual, leverage, and Cook's distance plots, as well as calculation of variance inflation factors. All models met these assumptions. Plots were created with the ggpubr and ggplot2 R packages.^31^, ^32^, ^33^

## 3 RESULTS

In cohort 1 (Norfrail), a total of 178 participants were included. Baseline characteristics of cohort 1 are given in Table 1, with further variables given in Appendix B. Details on diagnosis in the ED and which clinical departments the participants were admitted to are given in Appendix C. Study participants were 65 to 95 years old, and frailty increased with age (*p* < 0.001). Participants presented to the ED with a wide range of clinical scenarios across medical and surgical specialties. According to medical records, seven participants had previously been diagnosed with dementia, five of whom had a clinical diagnosis of AD. The distributions of frailty scores for CFS and mFFP are given in Appendix D.

**TABLE 1:** Cohort 1 (Norfrail) baseline characteristics.

Characteristic	Total, *n* = 178	Age 65 to 74, *n* = 76	Age 75 to 84, *n* = 76	Age ≥ 85, *n* = 26
Sex, *n* (%)				
Female	73 (41.0)	29 (38.2)	29 (38.2)	15 (57.7)
Male	105 (59.0)	47 (61.8)	47 (61.8)	11 (42.3)
Education, *n* (%)				
No formal education	1 (0.6)	0 (0)	0 (0)	1 (3.8)
Primary school	64 (36.0)	18 (23.7)	31 (40.8)	15 (57.7)
Secondary school	43 (24.2)	21 (27.6)	18 (23.7)	4 (15.4)
Higher education	70 (39.4)	37 (48.7)	27 (35.5)	6 (23.1)
Falls last year, *n* (%)	75 (42.1)	29 (38.2)	31 (40.8)	15 (57.7)
Polypharmacy^a^, *n* (%)	112 (62.9)	42 (55.3)	52 (68.4)	18 (59.2)
Modified Fried frailty phenotype, median (IQR)	1 (3)	1 (2)	2 (2)	3 (2)
Clinical Frailty Scale, median (IQR)	4 (3)	3 (2)	4 (3)	5 (2)
Mortality^b^, *n* (%)	11 (6.2)	2 (2.6)	5 (6.6)	4 (15.4)
Obese (*n* = 172), *n* (%)	31 (18.0)	16 (21.1)	13 (18.6)	2 (7.7)
Charlson Comorbidity Index, mean (SD)	5.20 (2.10)	4.41 (2.07)	5.58 (1.99)	6.42 (1.60)
Dementia^c^, *n* (%)	7^d^ (3.9)	1	2	4
eGFR (*n* = 177), mean (SD)	71.2 (23.7)	76.0 (23.3)	69.9 (22.5)	61.1 (25.5)
Plasma NfL, mean (SD) (*n*)	74.50 (95.20) [177]	51.45 (58.22) [76]	89.79 (124.12) [75]	97.79 (71.80)[26]
Plasma BD‐tau, mean (SD) (*n*)	21.35 (21.41) [177]	18.72 (24.30) [76]	22.70 (19.95) [75]	25.14 (15.33) [26]
Plasma UCHL1, mean (SD) (*n*)	162.11 (259.55) [173]	102.66 (125.49) [73]	202.17 (290.39) [74]	214.97 (392.32) [26]
Plasma GFAP, mean (SD) (*n*)	389.82 (378.48) [176]	305.29 (411.93) [76]	428.18 (359.65) [75]	531.70 (259.29) [25]
Plasma p‐tau217, mean (SD) (*n*)	1.84 (1.39) [172]	1.47 (1.06) [74]	1.85 (1.55) [73]	2.89 (1.21) [25]
Triage^e^ (*n* = 173), *n* (%)				
Green	6 (3.5)	0 (0)	4 (5.6)	2 (8.0)
Yellow	72 (41.6)	30 (39.5)	32 (44.4)	10 (40.0)
Orange	68 (39.3)	31 (40.8)	26 (34.2)	11 (44.0)
Red	27 (15.6)	15 (19.7)	10 (13.2)	2 (8.0)
Intensive care during hospital stay, *n* (%)	41 (23.0)	25 (32.9)	12 (18.4)	2 (7.7)

Abbreviations: BD‐tau, brain derived tau; eGFR, estimated glomerular filtration rate; GFAP, glial fibrillary acidic protein; IQR, interquartile range; *n*, number of participants; NfL, neurofilament light chain; p‐tau217, phosphorylated tau at threonine 217, SD, standard deviation; UCHL1, ubiquitin C‐terminal hydrolase L1.

^a^ Defined as use of five or more prescription medications.

^b^ Three‐month mortality.

^c^ Past medical condition.

^d^ Five of which were Alzheimer's disease, one vascular dementia, and one unspecified.

^e^ Triage in emergency department using Rapid Emergency Triage and Treatment System, where green is the least and red the most urgent.

In cohort 2, 105 individuals were identified and included: 34 CN and 71 living with MCI. Details of this cohort were described previously.^26^

### 3.1 NfL, but not BD‐tau or UCHL1, shows positive associations with frailty

**FIGURE 1:**
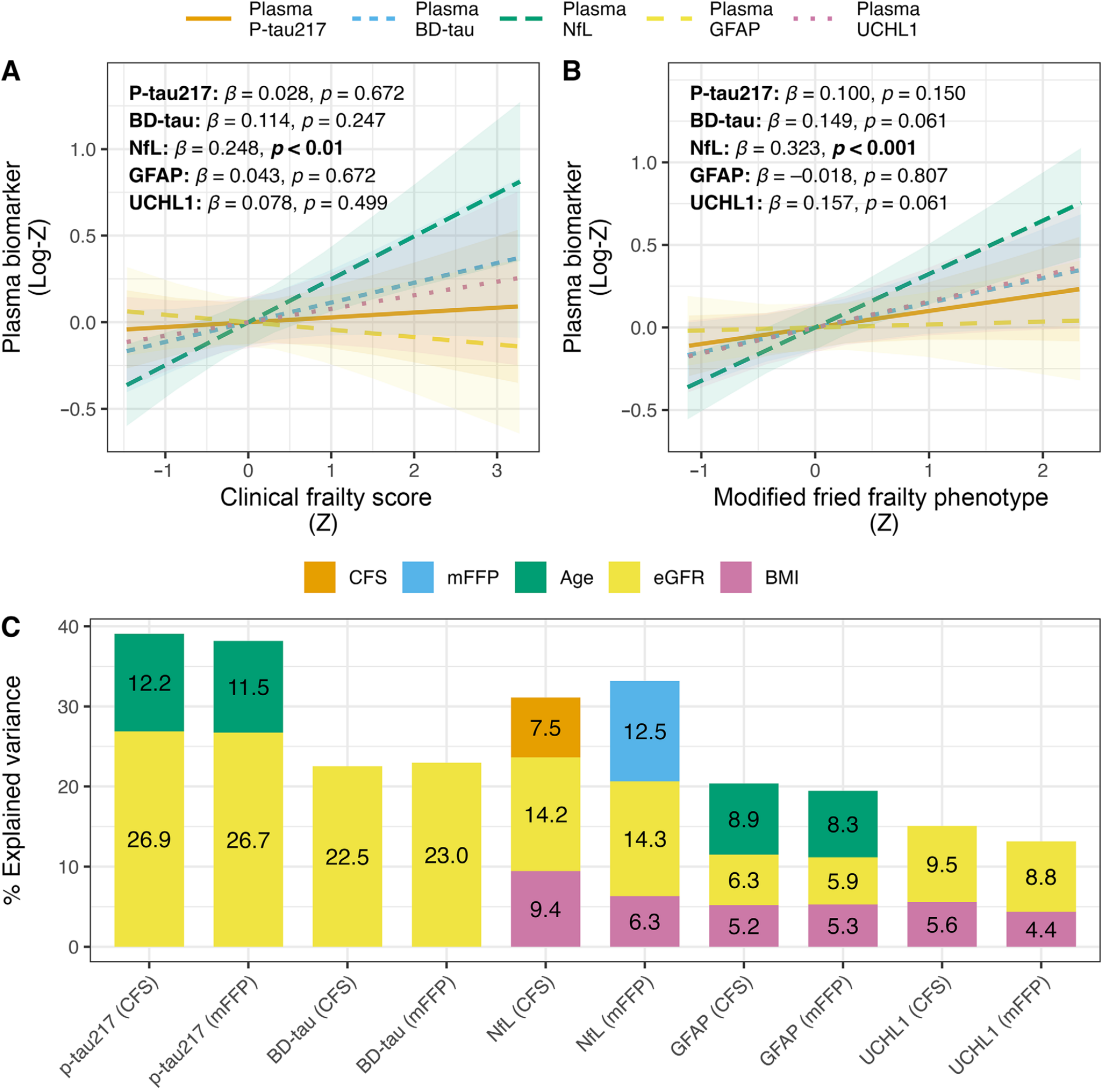
Associations between CFS, the mFFP, and plasma biomarkers. (A and B) Model‐predicted slopes for associations between CFS and mFFP, respectively, and each biomarker, adjusted for relevant covariates. The *y*‐axes represent log‐transformed and standardized (Z‐log) biomarker values, while the *x*‐axes represent standardized (Z) frailty scores. (C) Partial coefficients of determination (*R* ^2^_p_) for statistically significant predictors in each model. *R* ^2^_p_ values are multiplied by 100 to express the unique explained variance (%) for each predictor.

Both CFS (*β* = 0.248, *p* < 0.01) and mFFP (*β* = 0.323, *p* < 0.001) were associated with higher NfL concentrations, explaining 7.5% and 12.5% of the variance, respectively (Figure 1). Considering these results, we decided to conduct a subanalysis to address potential additional confounders. These included 4AT scores at time of blood sampling, the presence of acute brain injury, history of alcohol use, history of smoking, known dementia, previous history of stroke, transient ischemic attack, hypertension, and diabetes. The association remained (CFS: *β* = 0.250, *p* < 0.01; mFFP: *β* = 0.298, *p* < 0.001). In contrast, neither BD‐tau (CFS: *β* = 0.114, *p* = 0.247; mFFP: *β* = 0.149, *p* = 0.061) nor UCHL1 (CFS: *β* = 0.078, *p* = 0.499; mFFP: *β* = 0.157, *p* = 0.061) was significantly associated with frailty by either measure. Collectively, these findings suggest that frailty is more strongly reflected in neuroaxonal injury (NfL) than in the other injury/degeneration markers assessed in this study.

### 3.2 No association between GFAP and frailty

Adding to the mixed literature on GFAP and frailty, we found no evidence of an association with either frailty measure: CFS (*β* = −0.043, *p* = 0.672) or mFFP (*β* = 0.018, *p* = 0.807).

### 3.3 Plasma p‐tau217 does not associate with frailty

We observed no significant association between the plasma p‐tau217 and frailty after adjusting for covariates including age, sex, eGFR, CRP, and BMI. This finding was regardless of whether the CFS (*β* = 0.028, *p* = 0.672) or mFFP (*β* = 0.100, *p* = 0.150) was used to define frailty.

### 3.4 Pronounced associations between kidney dysfunction and p‐tau217 elevation

Across models, lower eGFR explained ∼27% of the variance in p‐tau217 and ∼23% in BD‐tau. Lower eGFR was also associated with higher NfL, UCHL1, and GFAP concentrations, but with smaller effect sizes (∼14%, ∼9%, and ∼6% explained variance, respectively) than observed for the tau markers. Higher BMIs were associated with lower NfL, UCHL1, and GFAP (∼8%, ∼5%, and ∼5% explained variance, respectively) but showed no association with the tau markers. Age was positively associated with p‐tau217 and GFAP, accounting for ∼12% and ∼8% of the variance, respectively. In contrast, NfL, BD‐tau, and UCHL1 were not associated with age and were therefore not retained in the final models during variable selection. Regression coefficients and effect sizes are provided in Figure 1 and Table 2.

**TABLE 2:** Linear regression of Clinical Frailty Scale (CFS) and modified Fried frailty phenotype (mFFP) with plasma biomarkers, adjusted for covariates.

Outcome	Predictors	Models with CFS	Models with mFFP
		*β* (95% CI), *R* ^2^_p_, ***p***	*β* (95% CI), *R* ^2^_p_, ***p***
P‐tau217	CFS	0.028 (−0.103, 0.159), *p *= 0.672^a^	
	mFFP		0.100 (−0.026, 0.226), *p *= 0.150^b^
	eGFR	−0.483 (−0.607, −0.359), *R* ^2^_p_ * *= 0.269, ***p *< 0.001**	−0.475 (−0.598, −0.353), *R* ^2^_p_ * *= 0.267, ***p *< 0.001**
	Age	0.312 (0.181, 0.443), *R* ^2^_p_ * *= 0.122, ***p *< 0.001**	0.293 (0.166, 0.42), *R* ^2^_p_ * *= 0.115, ***p *< 0.001**
BD‐tau	CFS	0.114 (−0.022, 0.249), *p *= 0.247^a^	
	mFFP		0.149 (0.016, 0.282), *p *= 0.061^b^
	eGFR	−0.476 (−0.611, −0.341), *R* ^2^_p_ * *= 0.225, ***p *< 0.001**	−0.474 (−0.607, −0.341), *R* ^2^_p_ * *= 0.230, ***p *< 0.001**
NfL	CFS	0.248 (0.114, 0.382), *R* ^2^_p_ * *= 0.075, ***p *< 0.01** ^a^	
	mFFP		0.323 (0.192, 0.455), *R* ^2^_p_ * *= 0.125, ***p *< 0.001**
	eGFR	−0.358 (−0.493, −0.223), *R* ^2^_p_ * *= 0.142, ***p *< 0.001**	−0.348 (−0.478, −0.217), *R* ^2^_p_ * *= 0.143, ***p *< 0.001**
	BMI	−0.276 (−0.407, −0.144), *R* ^2^_p_ * *= 0.094, ***p *< 0.001**	−0.219 (−0.349, −0.09), *R* ^2^_p_ * *= 0.063, ***p *< 0.001**
GFAP	CFS	−0.043 (−0.191, 0.105), *p *= 0.672^a^	
	mFFP		0.018 (−0.127, 0.163), *p *= 0.807^b^
	eGFR	−0.240 (−0.384, −0.097), *R* ^2^_p_ * *= 0.063, ***p *< 0.001**	−0.232 (−0.376, −0.089), *R* ^2^_p_ * *= 0.059, ***p *< 0.01**
	Age	0.310 (0.157, 0.464), *R* ^2^_p_ * *= 0.089, ***p *< 0.001**	0.290 (0.141, 0.439), *R* ^2^_p_ * *= 0.083, ***p *< 0.001**
	BMI	−0.213 (−0.354, −0.072), *R* ^2^_p_ * *= 0.052, ***p *< 0.01**	−0.215 (−0.356, −0.074), *R* ^2^_p_ * *= 0.053, ***p *< 0.01**
UCHL1	CFS^a^	0.078 (−0.07, 0.226), *p *= 0.499^a^	
	mFFP^b^		0.157 (0.01, 0.305), *p *= 0.061^b^
	eGFR	−0.309 (−0.457, −0.16), *R* ^2^_p_ * *= 0.095, ***p *< 0.001**	−0.293 (−0.439, −0.146), *R* ^2^_p_ * *= 0.0.88, ***p *< 0.001**
	BMI	−0.226 (−0.37, −0.081), *R* ^2^_p_ * *= 0.056, ***p *< 0.01**	−0.199 (−0.344, −0.054), *R* ^2^_p_ * *= 0.043, ***p *< 0.01**

*Note*: Ordinary least‐squares linear regressions with natural log‐transformed plasma biomarkers as dependent variables. For each biomarker, two models are shown with either CFS or mFFP as the primary predictor; additional covariates (estimated glomerular filtration rate [eGFR], body mass index [BMI], sex, or age) were retained according to the pre‐specified selection procedure. Standardized coefficients (*β*) with 95% confidence intervals (CIs) are reported. Partial coefficients of determination (*R* ^2^_p_) are presented for statistically significant predictors, representing the proportion of variance uniquely explained by each term. *P* values for the primary predictors of interest (CFS^a^ and mFFP^b^) were adjusted for multiple comparisons across biomarkers using the Benjamini–Hochberg method, whereas *p* values for covariates are unadjusted. Statistically significant values (*p* < 0.05) are highlighted in bold.

### 3.5 P‐tau217 comparison with DDI cohort (cohort 2)

**FIGURE 2:**
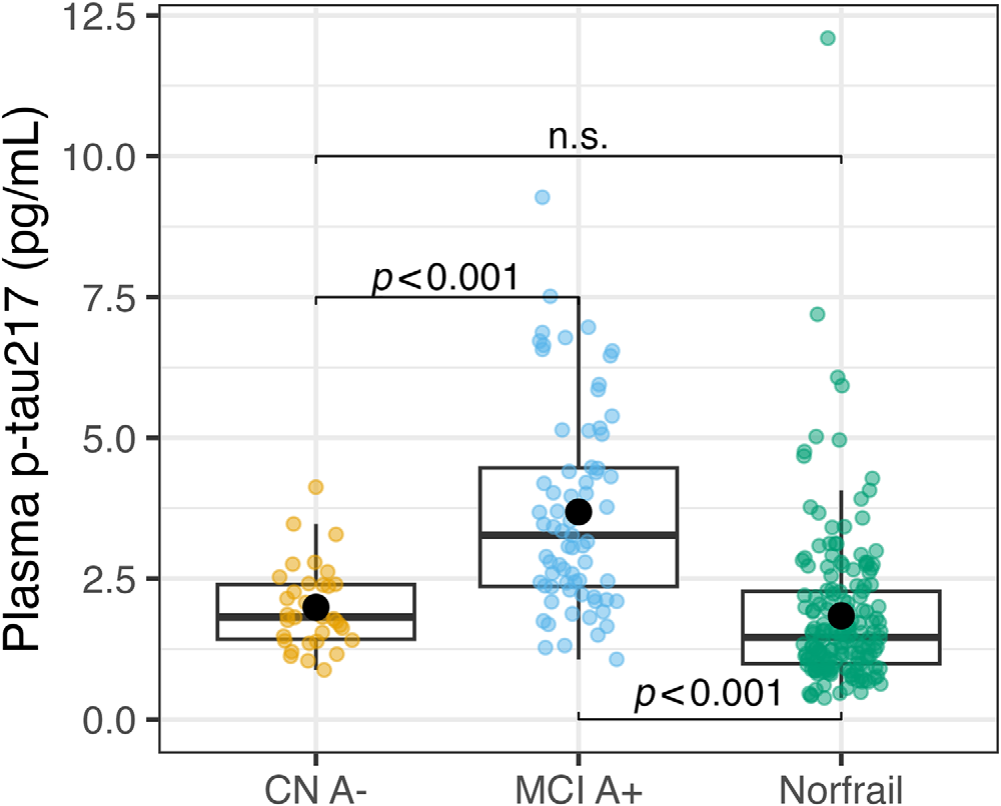
Between‐group comparisons of DDI cohort and Norfrail cohort. Cerebrospinal fluid Aβ42/40 ratio–defined amyloid‐positive (A+) and amyloid‐negative (A−) cases from DDI cohort are compared with participants from Norfrail cohort. Group means are indicated by black dots, and brackets denote post hoc pairwise comparisons. No adjustment was made for multiple comparisons.

In comparisons of biomarker levels from participants in cohorts 1 and 2, plasma p‐tau217 concentrations were significantly higher in MCI A+ compared with CN A− cases from the DDI cohort (Mdiff = 1.69, *p* < 0.001). However, cohort 1 (Norfrail) showed plasma p‐tau217 concentrations comparable to the CN A− group (Mdiff = 0.15, p = 0.580) and, consequently, a similar difference relative to the MCI A+ group (Mdiff = 1.66, *p* < 0.001) (Figure 2).

## 4 DISCUSSION

In this prospective study of older adults admitted acutely to hospital, we found no significant association between frailty and the AD‐associated plasma biomarkers p‐tau217, BD‐tau, UCHL1, and GFAP. Plasma NfL was the only biomarker significantly associated with frailty (*p* < 0.01), showing a positive linear relationship. The most striking finding in our study was the contribution of eGFR on p‐tau217 variance. This is clinically relevant as p‐tau217 is increasingly being utilized as a non‐invasive diagnostic AD biomarker, often involving older adults with impaired kidney function.

NfL was positively associated with frailty, confirming previous reports linking elevated NfL to frailty.^3^, ^15^, ^34^ NfL is a known marker of neuroaxonal degeneration, but originating not exclusively from the central nervous system. Other sites of origin may include peripheral neurons interacting with musculoskeletal tissue.^35^, ^36^, ^37^ We used the mFFP with assessment of grip strength as a measure for physical frailty.^2^ Others have shown a negative association between grip strength and NfL supporting a role for NfL in neuromuscular dysfunction.^38^, ^39^, ^40^ Moreover, plasma NfL is elevated in sarcopenia, a condition overlapping with frailty, defined by progressive loss of muscle mass and strength with neuronal and motor unit degeneration.^39^ Although distinct entities, raised NfL in frailty may prompt clinicians to assess for sarcopenia, a potentially reversible condition contributing to frailty.

Novelly, we report that the plasma biomarkers BD‐tau and UCHL1 are independent of frailty. Both proteins are associated with neuronal damage, with BD‐tau linked to AD. Elevated BD‐tau levels are found across a range of conditions, including traumatic brain injury, acute stroke, and AD.^41^, ^42^ Further, UCHL1 is also associated with traumatic brain injury and acute stroke.^43^, ^44^ Results in studies investigating GFAP and frailty are mixed.^16^, ^17^ In our study, we found no significant association between GFAP and frailty. Contrary to the hypothesis of a shared mechanism between biomarkers of neurocognitive disease and frailty, our findings support frailty and neurocognitive disease as separate entities. This is in line with a report on cerebrospinal fluid AD biomarkers including Aβ42 and p‐tau181, which did not differ with frailty status.^45^ Of note, BD‐tau, UCHL1, and GFAP are mainly found in the central nervous system, which may partly explain their lack of association with frailty.

P‐tau217, a core 1 biomarker in AD, is largely unexplored with regard to frailty. We used two separate frailty tools, mFFP and CFS, the latter a screening tool widely used in clinics and EDs.^6^ In contrast to our findings, a community‐based study reported an association between cognitive impairment, p‐tau217, and mFFP.^16^ This could be explained by different study populations, different background prevalences of cognitive impairment, or lack of adjustment for kidney function. The Norfrail cohort is heterogeneous, representing largely Caucasian older adults admitted acutely to hospital, the majority without known neurocognitive disease. Interestingly, for p‐tau217 we found levels comparable to those of healthy participants in the well‐characterized DDI cohort. This lends support to the idea that ADNC may be unrelated to the physical frailty syndrome and underlines the strong influence of AD pathology in p‐tau217 levels. Further, the use of a single biomarker may be inadequate to detect the multifactorial pathophysiology of frailty. Incorporating neurocognitive biomarkers in frailty assessment, such as NfL, p‐tau217, and BD‐tau, could potentially help to distinguish physical frailty from frailty driven by cognitive decline.

Of clinical importance, we note that eGFR explained over a quarter (26.9%) of the variance of p‐tau217 levels in our study. Kidney dysfunction has been reported to affect p‐tau217 levels in a few study populations, though not to the same extent as in our cohort.^46^, ^47^, ^48^, ^49^ In a recent Norwegian population‐based study examining p‐tau217, levels were affected when eGFR declined below 50 mL/min/1.73 m^2^, without affecting cut‐off levels.^48^ One explanation for the larger contribution of kidney dysfunction in our study includes participants with multimorbidity and a larger kidney function variability found in a hospital‐based cohort. Early AD biomarker cohorts include well‐stratified populations with less heterogeneity. This highlights the importance of validating biomarker findings in less‐selected cohorts where the effects of multimorbidity such as kidney function may become more evident.^50^, ^51^ In our study, eGFR does not represent the basal eGFR as it was measured only once during acute illness and acute hospital admission. Causes of a reduced eGFR include acute kidney injury but may equally represent chronic kidney disease. It is not clear how this affects p‐tau217 and is of interest for future exploration. Our findings of a large contribution of kidney dysfunction needs to be considered when interpreting p‐tau217 results. We suggest that p‐tau217 should be measured under clinically stable conditions, and not upon acute hospital admission. Clinicians should be cautious when using cut‐offs for AD in patients with chronic kidney disease.^49^

Of note, older age was associated with p‐tau217 and GFAP but not BD‐tau, NfL, or UCHL1. One explanation for this is that p‐tau217 and GFAP are thought to increase very early in AD, whereas BD‐tau, NfL, and UCHL1 increase in late‐stage disease due to progressive neurodegeneration.^52^ Another explanation for NfL may be that the frailty syndrome raises NfL levels to an extent where potential age association is masked. Furthermore, we found NfL, GFAP, and UCHL1 to be negatively associated with BMI, which is in line with other studies.^13^, ^53^, ^54^, ^55^, ^56^, ^57^ CRP did not significantly influence any of the ADNC biomarkers tested.

Like other studies from the ED, our cohort is heterogeneous with a wide spectrum of acute illnesses varying in severity and cause.^6^, ^58^ Transient functional decline due to acute illness may affect measures of physical frailty and plasma biomarkers. Although the CFS is defined by functional status 2 weeks prior to date of assessment, it does not account for illness lasting longer than 2 weeks.^7^ Other limitations include the absence of neurocognitive testing, which was not feasible in the acute ED setting and would likely not be representative of baseline cognitive function. Dementia comorbidity was assessed using patient records. It is therefore possible that participants with high biomarker levels represented undiagnosed or early‐stage neurodegenerative disease. In support of the low prevalence of dementia and MCI in our study, biomarker levels were comparable between cohort 1 and the cognitively healthy participants (A−) from cohort 2. Further, A+ MCI participants from cohort 2 had significantly higher levels of p‐tau217 compared with cohort 1. The clinical settings for recruitment between the cohorts differ significantly, and this needs to be taken into consideration when extrapolating results. Older adults living with severe frailty (CFS 7 to 9) were underrepresented in our study, and this was adjusted for in the regression analysis.

In conclusion, NfL was the only biomarker significantly associated with frailty in this study of unselected older adults admitted acutely to hospital. The AD biomarkers p‐tau217 and BD‐tau were independent of frailty status. Of clinical importance, p‐tau217 levels were highly affected by kidney dysfunction, and caution should be exercised when interpretingbiomarker results.

## CONFLICT OF INTEREST STATEMENT

Henrik Zetterberg has served on scientific advisory boards and/or as a consultant for AbbVie, Acumen, Alamar, Alector, Alzinova, ALZpath, Amylyx, Annexon, Apellis, Artery Therapeutics, AZTherapies, Cognito Therapeutics, CogRx, Denali, Eisai, Enigma, LabCorp, Merck Sharp & Dohme, Merry Life, Nervgen, Novo Nordisk, Optoceutics, Passage Bio, Pinteon Therapeutics, Prothena, Quanterix, Red Abbey Labs, reMYND, Roche, Samumed, ScandiBio Therapeutics AB, Siemens Healthineers, Triplet Therapeutics, and Wave, has given lectures sponsored by Alzecure, BioArctic, Biogen, Cellectricon, Fujirebio, LabCorp, Lilly, Novo Nordisk, Oy Medix Biochemica AB, Roche, and WebMD, is a co‐founder of Brain Biomarker Solutions in Gothenburg AB (BBS), which is a part of the GU Ventures Incubator Program, and is a shareholder of CERimmune Therapeutics (outside submitted work). The other authors declare no conflicts of interest. Author disclosures are available in the Supporting Information.

## Supporting information




Supporting Information

Supporting Information

## Data Availability

Due to participant confidentiality, it is not possible to share our datasets. Upon request, collaborations are possible, subject to ethics review board and data protection officer approval. All study participants provided written informed consent. In cases of impaired mental capacity, the participants’ guardians were also consulted before inclusion.
