# Supplementary material for: Interpreting p‐tau217 on the ward: frailty and plasma biomarkers in acutely admitted older adults
Source: Alzheimers Dement. 2026 Aug 21;22(8):e71768. doi: 10.1002/alz.71768 (PMC13494674; doi:10.1002/alz.71768)
Supplement: Supplementary file 2 [file ALZ-22-e71768-s002.docx]

## Appendix

**Appendix A. Selection of participants from the DDI cohort for this study:**

The DDI cohort initially included 110 CN participants with normal amyloid status, defined by CSF Aβ_42/40_ ratios (CN A-), and 113 MCI participants with pathological Aβ_42/40_ ratios (MCI A+), all of whom had available plasma p-tau217 measurements. However, the DDI cohort is younger than the Norfrail sample. To reduce potential bias from age differences between cohorts, subsamples of CN A- and MCI A+ participants from the DDI cohort were frequency-matched to the Norfrail sample. This resulted in 34 CN A- and 71 MCI A+ cases selected for comparison with the Norfrail sample. While age matching between the DDI cohort and the Norfrail sample was attempted, residual age differences remained after matching. Participants in the Norfrail cohort were older on average (M*=*76.42, *SD*=7.23) compared to both CN A- (*M*=71.44, *SD*=3.27 years) and MCI A+ (*M*=72.93, *SD*=4.15 years) groups. The difference between CN A- and MCI A+ was not statistically significant (*p*=.252), whereas both DDI groups differed significantly from the older Norfrail sample (*p*<0.001). The CN A− and MCI A+ groups comprised 14 (41%) and 35 (49%) females, respectively, whereas the Norfrail sample included 73 (41%) females. No significant differences in sex distribution were observed (*χ^2^* = 1.48, p = 0.477).

**Appendix B. Additional baseline characteristics for the Norfrail cohort.**

| Characteristic | Total, n = 178 | Age 65-74, n = 76 | Age 75-84, n = 76 | Age ≥ 85, n = 26 |
| --- | --- | --- | --- | --- |
| History of smoking, n (%) | 119 (66.9) | 53 (69.7) | 53 (69.7) | 13 (50.0) |
| History of alcohol use, n (%) | 161 (90.4) | 73 (96.1) | 65 (88.2) | 21 (80.8) |
| Hypertension*, n (%) | 111 (62.4) | 44 (57.9) | 52 (68.4) | 15 (57.7) |
| Diabetes*, n (%) | 27 (15.2) | 13 (17.1) | 12 (15.8) | 2 (7.7) |
| Stroke and/or transient ischemic attack*, n (%) | 34 (19.1) | 12 (15.8) | 15 (19.7) | 7 (26.9) |
| CKD Stage 3 or higher*, n (%) | 37 (20.8) | 12 (15.8) | 14 (18.4) | 11 (42.3) |
| CRP, mean (SD) | 28.3 (55.4) | 22.1 (49.6) | 37.8 (64.9) | 19.0 (35.8) |
| Blood sample timing†, mean (SD) | 13:33 (6:06) | 13:23 (6:09) | 14:02 (6:03) | 12:42 (6:15) |
| 4AT at time of blood sampling‡, n (%) | 13 (7.3) | 1 (1.3) | 9 (11.8) | 3 (11.5) |
| Acute brain injury§, n (%) | 12 (6.7) | 5 (6.6) | 4 (5.3) | 3 (11.5) |

n: number of participants. IQR: interquartile range. SD: standard deviation. CKD: Chronic kidney disease. CRP: C-Reactive Protein. *: Past medical condition. †: Time given in hours:minutes between participant arriving at hospital and venous blood sampling for biomarkers. ‡: given in number of participants with 4AT score of 4 or higher. §: participants admitted due to ischemic stroke, transient ischemic attack, cerebral haemorrhage or head injury.

**Appendix C1. Emergency department discharge diagnosis in Norfrail cohort**
Number of participants (n) with each diagnosis is given in the column to the right of the code.

| **Diagnosis** | **n (%)** |
| --- | --- |
| Injury | 18 (10.2) |
| Fever | 19 (10.7) |
| Dyspnoea | 15 (8.5) |
| Chest pain | 42 (23.7) |
| Palpitations | 13 (7.3) |
| Heart failure | 9 (5.1) |
| Abdominal pain | 12 (6.8) |
| Pneumonia | 11 (6.2) |
| Vertigo | 2 (1.1) |
| Syncope | 6 (3.4) |
| Neurological deficits | 12 (6.8) |
| Other | 18 (10.2) |

**Appendix C2. Participant distribution by medical field in Norfrail cohort**
Number of participants (n) is given in the column to the right of the medical field.

| **Medical field** | **n (%)** |
| --- | --- |
| Cardiology | 71 (39.9) |
| Pulmonology | 21 (11.8) |
| Infectious diseases | 11 (6.2) |
| Nephrology | 6 (3.4) |
| Gastroenterology | 4 (2.2) |
| Hematology | 8 (4.5) |
| Geriatrics | 5 (2.8) |
| Rheumatology | 1 (0.6) |
| Dermatology | 1 (0.6) |
| Otolaryngology | 1 (0.6) |
| Gastrointestinal surgery | 10 (5.6) |
| Urology | 2 (1.1) |
| Cardiothoracic surgery | 2 (1.1) |
| Neurosurgery | 6 (2.4) |
| Orthopedics | 15 (8.4) |
| Neurology | 2 (2.2) |
| Stroke unit | 9 (5.1) |
| Oncology | 1 (0.6) |

**Appendix D. Frailty distribution in the Norfrail cohort:**

Number of participants

| Clinical Frailty Scale score | n = |
| --- | --- |
| 1 | 20 |
| 2 | 42 |
| 3 | 23 |
| 4 | 35 |
| 5 | 36 |
| 6 | 15 |
| 7 | 5 |
| 8 | 0 |
| 9 | 2 |

**Appendix D.1: distribution of Clinical Frailty Scale scores in the Norfrail cohort.** Number of participants (n) for each Clinical Frailty Scale score is given within each column.

Number of participants

| Modified Fried Frailty score | 0,1,2,3,4,5 |
| --- | --- |
| 0 | 47 |
| 1 | 45 |
| 2 | 36 |
| 3 | 28 |
| 4 | 14 |
| 5 | 8 |

**Appendix D.2: distribution of modified Fried Frailty scores in the Norfrail cohort.** Number of participants (n) for each modified Fried Frailty score is given within each column.
